# Preserving stroke penumbra by targeting lipid signalling

**DOI:** 10.1177/0271678X221121853

**Published:** 2022-08-23

**Authors:** Beatriz Achón Buil, Ruslan Rust

**Affiliations:** 1 Institute for Regenerative Medicine, University of Zurich, Schlieren, Switzerland; 2 Neuroscience Center Zurich, University of Zurich and ETH Zurich, Zurich, Switzerland

**Keywords:** Autotaxin, hyperexcitability, ischemia, penumbra, therapy

## Abstract

Pharmacological inhibition of astrocytic enzyme autotaxin rescues the stroke penumbra in mice and improves functional recovery, indicating therapeutic potential.

Ischemic stroke is a leading cause of disability and death; and treatment options for patients are very limited. The sudden reduction of oxygen and nutrients triggers an irreversible damage and necrosis in the ischemic core. Interestingly, the area surrounding the infarction, termed penumbra, forms a region of electrical silence in initially intact but hypoxic tissue. The presence of deleterious metabolic processes, such as excitotoxicity, leads to the conversion of penumbra into ischemic core over time if there is no therapy initiated.^1^ Previous attempts of therapeutic excitotoxicity blockade e.g., antagonists of N-methyl-D-aspartate receptor (NMDAR) have all failed,^2^ mainly because of the unintended blockade of physiological glutamate transmission. Therefore, a specific targeting of pathological glutamate transmission needs to be addressed.

Astrocytes are involved in the regulation of glutamatergic transmission via the autotoxin (ATX) and lysopophosphatidic acid (LPA) lipid signalling. ATX is an enzyme that converts lysophosphatidylcholine (LPC) into the bioactive LPA. Astrocytic ATX release is regulated by glutamate, and it takes place specifically in excitatory but not inhibitory synapses.^3^ LPA interacts with LPA_2_ receptor in the presynaptic neuron leading to an increased probability of glutamate release. LPA uptake is regulated by plasticity related gene-1 (PRG-1), which is located in the postsynaptic membrane on glutamatergic neurons. ATX/LPA axis dysregulations are associated with neurological disorders such as neuropathic pain, spinal cord injury, stroke, multiple sclerosis and psychiatric disorders.^3,4^

Writing in *Science Translation Medicine*, Bitar, Uphaus, Thalman and colleagues hypothesized that ATX/LPA axis dysregulation post-stroke leads to hyperexcitability and inhibiting this pathway could be a promising therapeutic strategy to rescue the penumbra (Figure 1).

**Figure 1.**
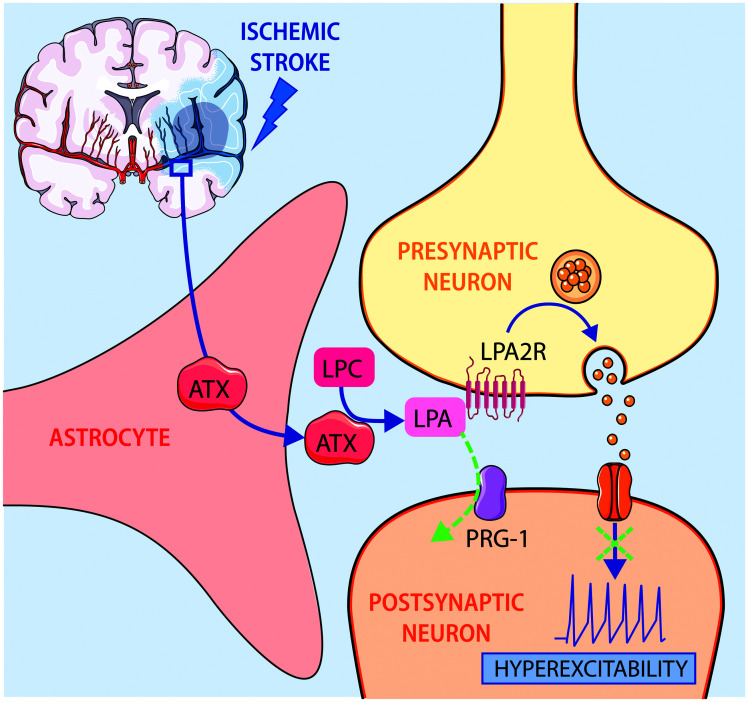
Autotaxin (ATX) release by astrocytes is increased after ischemic stroke, resulting in higher levels of lysophosphatidic acid (LPA) which in turn rise the probability of glutamate release leading to hyperexcitability in the penumbra region. This signalling pathway is negatively regulated via LPA uptake by plasticity-related gene 1 (PRG-1).

First, the authors describe an unprecedent role of astrocytes in penumbra expansion. Previous studies have focused on astrocytic release of neurotrophic factors or inflammatory molecules,^5^ whereas this study demonstrate the direct involvement of astrocytes in synapse modulation leading to hyperexcitability and eventually worse stroke outcome. Protein levels of ATX and glial fibrillary acidic protein (GFAP), which is a marker for reactive astrocytes, were increased and found close to each other at 72 h after middle cerebral artery occlusion (MCAO) in mice. Then, the authors used a mouse model with specific ATX deletion in reactive astrocytes (*Atx^fl/fl^ Gfap-Cre^+^*mice) which showed improved functional recovery (measured with modified neurological severity score (mNSS)), and reduced infarct volume and caspase-3 induced apoptosis 72 h post-stroke.

Second, the authors investigated ATX, LPA and LPC concentrations in human cerebrospinal fluid (CSF) samples. Higher concentrations of CSF ATX were observed up to 14 days post-stroke, which correlated with higher perfusion mismatch and worse stroke outcome, measured by the National Institutes of Health Stroke Scale (NIHSS) score. Mass spectrometry analysis revealed that also LPA and LPC CSF levels were higher in stroke patients compared to age- and sex-matched controls. High LPA levels were also found in the CSF of MCAO mice, which had been previously associated with excitatory/inhibitory balance dysregulation.^3^

To better understand the role of lipid signalling in stroke outcome, the authors studied individuals carrying a Single Nucleotide Polymorphism (SNP) in PRG-1 (PRG-1^R345T^), that was described to prevent the LPA uptake.^6^ Patients carrying the SNP presented a higher NIHSS at 24 h post-stroke. The analogue mouse model (PRG-1^R346T^) also presented a higher infarct volume and worse neurological outcomes (mNSS). These mice showed higher levels of activated caspase-3^+^ cells in the peri-infarct regions and increased neurofilament light (NfL) chain concentrations in the CSF, which is associated with neuronal damage and stroke mortality. Besides, the PRG-1^R346T^ mouse model showed more spontaneous glutamatergic events suggesting a higher excitability than PRG-1^WT^ mice.

To determine the translational potential of lipid signalling blockade, the authors administered a small molecule (PF8380) that selectively prevent the LPA synthesis function of ATX. Pharmacological inhibition was carried out in stroked PRG-1^WT^ and PRG-1^R346T^ mice, by daily intraperitoneal injection of PF8380 up to 72 h. Electrophysiological assays showed that enhanced spontaneous excitatory postsynaptic currents (sEPSC) and increased cortical neuron firing following MCAO can be reduced by PF8380 administration in both strains. Furthermore, ATX inhibition resulted in lower apoptosis, reduced infarct volume and better mNSS score in PRG-1^WT^ and PRG-1^R346T^ mice.

In order to determine the therapeutic window, the effect of PF8380 was analysed at 30, 60, 90 and 180 min post-stroke. Already after 3 h there was an improvement in functional outcome and infarct volume when ATX inhibitor was administered up to 90 min post-MCAO.

The main limitations of the study are due to the small patient cohort and the use of animal models. Stroke was only modelled by inducing transient MCAO via the intraluminal suture technique, and thus, it may be reasonable to validate the results in a second stroke model. Furthermore, PF8380 is administered intraperitoneally which is not the preferred route for humans, and its delivery across the blood-brain barrier might entail a challenge for clinical translation. Additionally, the ratio of neurons to astrocytes in humans differs from that in mice, which may affect translation to patients. For instance, clinical trials targeting other astrocytic secreted proteins after stroke e.g., S100-β, have shown mixed results in translating promising preclinical findings.^5^ Therefore, a broader understanding of the astrocyte role in stroke should be considered. For example, higher levels of endocannabinoids following electroacupuncture pre-treatment upregulated extracellular glutamate and improved stroke outcomes in a MCAO mouse model, via astrocytic cannabinoid type 1 receptors (CB_1_R).^7^ Moreover, astrocytes are directly involved in regulating cerebral blood flow via vasoactive substance secretion,^8^ which is crucial for rescuing the penumbra. Future studies may also perform more in-depth behavioural analysis to gain more detailed insights into the improved functional recovery^9^ and may combine ATX inhibitors with other promising regenerative strategies currently being tested in preclinical mouse models.^10^

Overall, Bitar, Uphaus, Thalman and colleagues demonstrated the involvement of lipid signalling in stroke outcome by different approaches (cell-type specific deletion, genetic models, and pharmacological inhibition) which allows validation of ATX as a potential target for stroke treatment. ATX inhibition in MCAO mice decreased the pathological hyperexcitability without affecting the physiological glutamate transmission. Therefore, these relevant findings can have a direct translation into clinic for savaging the penumbra and improving stroke outcome in future.
